# A case of iatrogenic transmission of vascular Aß40 amyloid

**DOI:** 10.1002/alz70857_097361

**Published:** 2025-12-24

**Authors:** Takeshi Kawarabayashi, Takumi Nakamura, Shin Takatama, Naoko Miyamoto, Tomoyuki Iwai, Isao Naito, Takashi Sugawara, Kunihiko Ishizawa, Kentaro Hashimoto, Masakuni Amari, Takeshi Ikeuchi, Hiroo Kasahara, Yoshio Ikeda, Masamitsu Takatama, Mikio Shoji

**Affiliations:** ^1^ Geriatrics Research Institute and Hospital, Maebashi, Japan; ^2^ Gunma University Graduate School of Medicine, Maebashi, Japan; ^3^ Geriatrics Research Institute and Hospital, Maebashi, Gunma, Japan; ^4^ Geriatrics Research Institute and Hospital, Maebashi, Gunma, Japan; ^5^ Brain Research Institute, Niigata University, Niigata, Japan; ^6^ Gunma University Gradutae School of Medicine, Maebashi, Gunma, Japan; ^7^ Gunma University, Maebashi, Gunma, Japan

## Abstract

**Background:**

Iatrogenic cerebral amyloid angiopathy (iCAA) has recently been reported in relatively young adults who received cadaveric dural grafts or neurosurgery during childhood. More than 80 cases of iCAA have been reported; however, insufficient detail regarding Aß40 amyloid transmission was provided in these reports.

**Method:**

We present a case of Aß iCAA, which showed a unique clinical course 33 years after a cadaveric dural graft and fulfilled all of the proposed criteria.

**Result:**

A 42‐year‐old man developed a subcortical intracranial hemorrhage (ICH) in the right temporoparietal lobe. He had undergone the surgical removal of an eosinophilic granuloma in the right frontal bone and grafting with LYODURA cadaveric dura at the age of 9 years. Susceptibility‐weighted images of MRI taken at 38 years old showed multiple incidental microbleeds (MBs) in the right frontal lobe around the LYODURA. At 18 and 19 months after the first ICH, he suffered a 2nd (in the right parietal lobe) and 3rd (in the right frontal lobe) ICH, respectively. The brain tissue in the removed hematoma predominantly showed Aβ40 deposition in the leptomeningeal vessels. No neurofibrillary tangles or neuropil threads were noted. Genetic testing revealed no *APP or PSEN‐1/‐2* mutations, and the *APOE* genotype was ε3/ε3. The cerebrospinal fluid (CSF) levels of Aβ42 and Aβ40 were decreased, but those of total tau and phosphorylated tau181 were within the normal range. Corticosteroid therapy was administered to prevent further ICHs. Five months later, follow‐up CT showed a 4th incidental ICH in the right medial frontal cortex though It was small and asymptomatic. During 2 years’ follow‐up no further ICHs or symptoms appeared.

**Conclusion:**

Our case is suggested to be a precisely described probable case of vascular Aß40 amyloid transmission and propagation from LYODURA. Corticosteroid therapy may be effective at preventing further Aß iCAA‐induced ICHs though there is no evidence of a beneficial effect in non‐inflammatory iCAA.

